# Optimised Motion Tracking for Positron Emission Tomography Studies of Brain Function in Awake Rats

**DOI:** 10.1371/journal.pone.0021727

**Published:** 2011-07-01

**Authors:** Andre Z. Kyme, Victor W. Zhou, Steven R. Meikle, Clive Baldock, Roger R. Fulton

**Affiliations:** 1 School of Physics, University of Sydney, Sydney, Australia; 2 Brain and Mind Research Institute, University of Sydney, Sydney, Australia; 3 Faculty of Health Sciences, University of Sydney, Sydney, Australia; 4 Department of Medical Physics, Westmead Hospital, Sydney, Australia; 5 School of Engineering and Built Environment, University of Central Queensland, Mackay, Australia; University of Texas, M.D. Anderson Cancer Center, United States of America

## Abstract

Positron emission tomography (PET) is a non-invasive molecular imaging technique using positron-emitting radioisotopes to study functional processes within the body. High resolution PET scanners designed for imaging rodents and non-human primates are now commonplace in preclinical research. Brain imaging in this context, with motion compensation, can potentially enhance the usefulness of PET by avoiding confounds due to anaesthetic drugs and enabling freely moving animals to be imaged during normal and evoked behaviours. Due to the frequent and rapid motion exhibited by alert, awake animals, optimal motion correction requires frequently sampled pose information and precise synchronisation of these data with events in the PET coincidence data stream. Motion measurements should also be as accurate as possible to avoid degrading the excellent spatial resolution provided by state-of-the-art scanners. Here we describe and validate methods for optimised motion tracking suited to the correction of motion in awake rats. A hardware based synchronisation approach is used to achieve temporal alignment of tracker and scanner data to within 10 ms. We explored the impact of motion tracker synchronisation error, pose sampling rate, rate of motion, and marker size on motion correction accuracy. With accurate synchronisation (<100 ms error), a sampling rate of >20 Hz, and a small head marker suitable for awake animal studies, excellent motion correction results were obtained in phantom studies with a variety of continuous motion patterns, including realistic rat motion (<5% bias in mean concentration). Feasibility of the approach was also demonstrated in an awake rat study. We conclude that motion tracking parameters needed for effective motion correction in preclinical brain imaging of awake rats are achievable in the laboratory setting. This could broaden the scope of animal experiments currently possible with PET.

## Introduction

Positron emission tomography (PET) is a non-invasive imaging technique that uses positron-emitting radioisotopes to study functional processes within the body. PET measurements provide information about the spatial distribution and expression levels of specific cellular targets such as receptors or enzymes. Changes in functional activity due to physiological, pathological or pharmacological challenges are also readily measured. Small animal PET, characterised by smaller scanner bore size and crystal size, and higher spatial resolution compared with human PET, now plays a key role in preclinical research based on animal models. Here the ability to perform longitudinal studies in the same animal is particularly useful [1]. A comprehensive description of PET physics, instrumentation and methodology can be found in [2].

Laboratory animals undergoing in-vivo brain imaging procedures are normally anaesthetised to eliminate both stress and movement. In some countries the use of anaesthesia to minimise stress is mandatory. However, there are two important drawbacks of sedation which limit the potential of PET. Firstly, the literature contains numerous examples of anaesthetic drugs affecting physiological measurements in the brain (e.g. auditory response [3], radioligand binding [4], glucose metabolism [5], cerebral blood flow [6], motor-evoked potentials [7], neural activity [8], neuro-hemodynamic coupling [9] and neurotransmitter flux [10]). In each case, the signal of interest was either masked, inhibited, or exaggerated with respect to anaesthetic-free controls. Secondly, use of anaesthesia prevents investigators from performing imaging studies on freely moving animals during normal and evoked behaviours, meaning that at present many rich experimental paradigms to elucidate the neurological response to external stimuli in animals cannot be exploited. These are significant limitations given that PET is currently the only non-invasive method to study specific biological correlates of behaviour (i.e. neurochemical or receptor changes).

Therefore, overcoming the need to use anaesthetic drugs during imaging experiments has been recognised as an important research objective. Apart from physically restraining the animal (eg. [5], [9], [11], [12]), which can readily stress the subject (eg. [13], [14]), a motion compensation approach is generally adopted (one exception is [15]). Motion compensation refers to a general methodology whereby the subject's three-dimensional (3D) head motion is measured during the study and subsequently accounted for before or during image reconstruction. This has been demonstrated in SPECT of mice using a stereo-optical setup to determine the motion of retro-reflective markers glued to the head [16].

**Figure 1:**
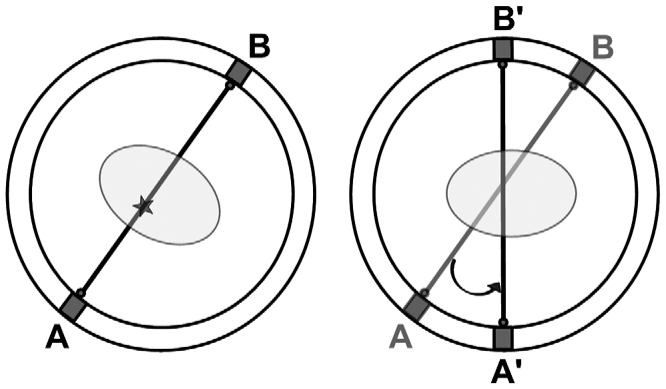
Principle of line-of-response (LOR) rebinning. A LOR represents the detected path of a pair of coincident gamma photons. The left side of the figure shows an LOR detected on the line joining detectors A and B when the object (ellipse) was in a certain (moved) pose. The right side shows the original object pose, and indicates how the LOR ought to be reoriented so as to correctly coincide with this pose i.e. it now lies on A′B′. Notice that in both cases the path through the object is the same. Although shown here in 2D, the transformations are 3D in general.

The approach we have developed for rats undergoing PET brain scans also uses a stereo-optical setup for rigid-body motion tracking. Correction is performed using a strategy that was developed originally for human PET scanning [17]. In this method, each line of response (LOR) representing a detected coincidence is spatially transformed according to the measured motion prior to being reconstructed [17]–[19] (see figure 1). Previously we have applied these methods to correct for step-wise motion in phantom studies performed on a small animal PET scanner [20]. Our goal, however, is to enable correction of continuous motion, which is both rapid and arbitrary, as is expected when imaging conscious animals. Due to the frequent and rapid motion exhibited by alert, awake small animals, optimal motion correction requires frequent sampling of the subject's pose and precise synchronisation of the pose measurements with events in the PET coincidence data stream. Inadequacies in either of these areas are expected to reduce the qualitative and quantitative accuracy of image-based measurements. In addition, to avoid degrading the excellent spatial resolution provided by state of the art small animal PET scanners, the motion measurements themselves should be as accurate as possible.

In this study, our aim was to establish the feasibility of obtaining quantitatively accurate motion-corrected images of the awake rat brain using PET and suitably optimised motion tracking techniques. We describe a hardware-based synchronisation approach with low latency and high accuracy and validate it in a range of phantom studies involving manually applied arbitrary continuous movements, as well as rat motion applied using a six-axis robot. We also explored the impact of synchronisation error and pose sampling rate on the quality and quantitative accuracy of motion-compensated images, as well as the effect of marker size and rate of object motion. Our results demonstrate the relative impact of these various factors, and also indicate that motion tracking parameters needed for effective motion correction in preclinical brain imaging of awake rats are achievable in the laboratory setting. This could broaden the scope of animal experiments currently possible with PET.

## Materials and Methods

### 1. Ethics Statement

All animal work described was conducted in accordance with a protocol approved by the Animal Ethics Committee of the University of Sydney (Protocol Number: K00/12-2008/2/4891) and with the Australian Code of Practice for the Care and Use of Animals for Scientific Purposes 7th Edition 2004.

### 2. Data Acquisition

PET measurements were performed on a microPET Focus 220 small animal PET scanner (Preclinical Solutions, Siemens Healthcare Molecular Imaging, Knoxville, TN, USA). The microPET comprises 168 lutetium oxyorthosilicate (LSO) detectors arranged in 4 contiguous rings. Each detector is a 12×12 LSO pixelated array of crystal elements of dimension 1.51×1.51×10 mm^3^. The field of view (FOV) is 19 cm in the transaxial (x–y) direction and 7.6 cm in the axial (z) direction. Reconstructed image resolution at the centre of the FOV is 1.3 mm [21]. All data were acquired in list mode format.

### 3. Motion Tracking

Motion measurements were obtained using a stereo-optical motion tracker (MicronTracker Sx60, Claron Technology Inc., Toronto, Canada). The device collects two slightly offset images of a scene many times per second. These stereo ‘frames’ enable the pose (position and orientation) of specially designed markers in its field of measurement (FOM) to be computed at a rate of up to 48 Hz. A general purpose I/O (GPIO) interface allows it to be externally triggered using TTL pulses and/or to transmit TTL pulses as poses are acquired. We refer to the latter as pulse strobing. A more detailed characterisation of the MicronTracker and marker design can be found in [22].

**Figure 2:**
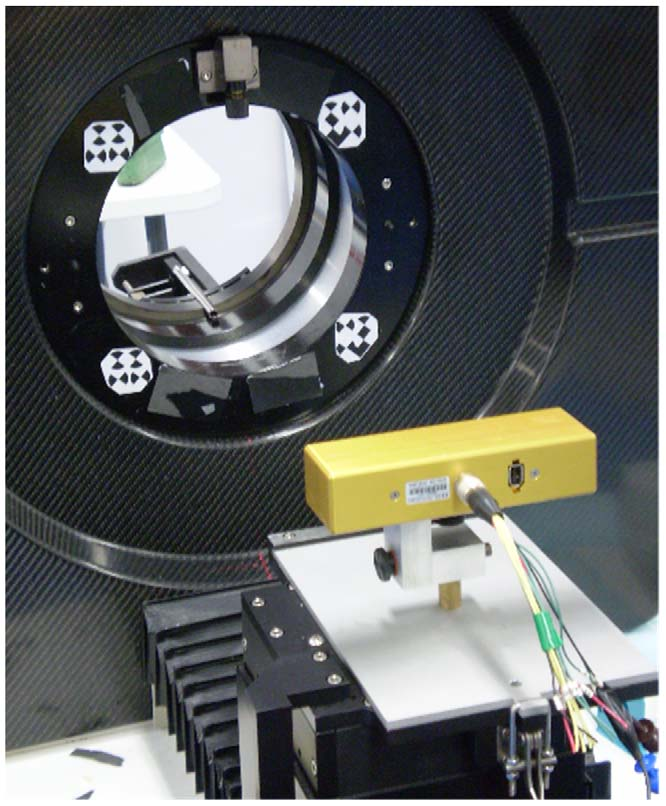
Setup for motion tracking. The MicronTracker stereo tracking system is shown here in the foreground attached to the scanner bed unit via a custom-made mount. In the background is the microPET scanner bore. A 4-part marker affixed to the scanner bore is used to define a reference coordinate system and enables changes in the relative pose of the tracker and scanner to be accounted for.

Figure 2 shows the motion tracking system as used in the microPET environment. The tracker was positioned 50 cm from the centre of the microPET field of view (FOV). At this distance the tracker FOM included the microPET FOV and a reference marker affixed to the gantry (figure 2). A calibration of the tracker and scanner coordinate systems was performed to enable tracker pose measurements to be converted to the PET coordinate system [22]. The gantry reference marker enabled the calibration to be adjusted according to changes in the relative position of the tracker and scanner.

**Figure 3:**
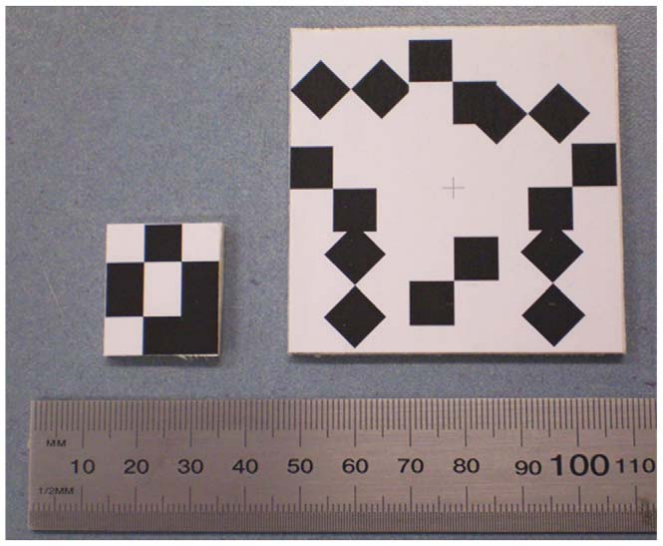
Markers for tracking. The small 3-point marker (left) suitable for rat head tracking, and the larger 8-point marker (right) used as a performance reference. A minimum of three X-points (intersections of black and white regions) are required for six degree-of-freedom pose tracking. The 8-point marker therefore has considerable redundancy for pose estimation.

We designed a 3-point marker, of mass 0.6 g and dimensions 24 mm×21 mm, for use in awake rat studies. It was small enough to be attached to a rat's head without touching the ears or obstructing its vision, which could stress the animal. For comparison we also used a larger marker (60 mm×60 mm) comprising eight points arranged radially. Both markers are shown in figure 3.

A minimum of 3 points are required to establish rigid pose. MicronTracker target points (known as “Xpoints”) correspond to the intersection of the surrounding black/white target regions (figure 3) and can only be detected if the projection footprint of the target region on the CCD sensor exceeds a manufacturer-defined threshold. Larger target regions enable a marker to be detected over a greater angular range. The specified positional accuracy for an Xpoint is 0.25 mm RMS. The 8-point marker used in this study had a greater range of angular detection than the 3-point marker (by virtue of its larger target regions), greater redundancy (due to the greater number of target points), and better overall accuracy (due to the target points being further apart and more symmetrically placed) [23].

### 4. Synchronisation

**Figure 4:**
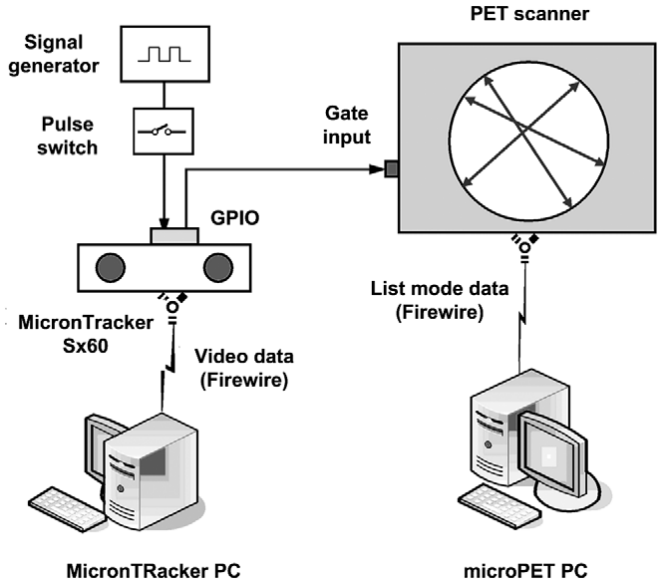
Hardware setup for synchronisation of tracker and list mode PET data. Acquisition of time-stamped stereo image frames is initiated and controlled by a signal generator triggering the tracker's external input at the desired frequency. With each frame the tracker triggers the gating input of the microPET, initiating the insertion of a data ‘tag’ in the list mode event stream.

Synchronisation of the tracker and scanner data streams was performed by inserting a data tag into the PET list mode stream at the time of each pose measurement. As shown in figure 4, the MicronTracker was externally triggered via the GPIO interface at a frequency *f* by pulse waves from a signal generator. The falling edge of each trigger pulse initiated both the exposure of a tracker stereo image frame for a shutter time *T_E_* ms and, simultaneously, a strobe output pulse of duration *T_S_* ms. The strobe output was connected to the gating input of the scanner to trigger the insertion of tags in the PET list mode data. Stereo images acquired by the tracker were transferred to a PC and processed to extract marker pose parameters using software provided by the manufacturer. After acquisition, inserted tags were associated with tracker pose measurements in two stages: pose-tag matching and temporal alignment.

#### 4.1. Pose-tag matching

Triggering was stopped just before the end of the microPET scan to enable the last tracker measurement to be reliably identified. This was because not all pulses reaching the microPET gating input caused tags to be inserted in the list mode data. It is not clear why this occurred, but we observed that the majority of dropped tags occurred within the first few seconds of an acquisition. Dropped tags were identified and accounted for using the time intervals between successive tags based on the regular 1 ms time marks in the list mode data. It was noted that although dropped tags occurred in most scans, in approximately 50 trials they represented a negligible proportion (<0.05 %) of the study time in all cases.

#### 4.2. Temporal alignment

**Figure 5:**
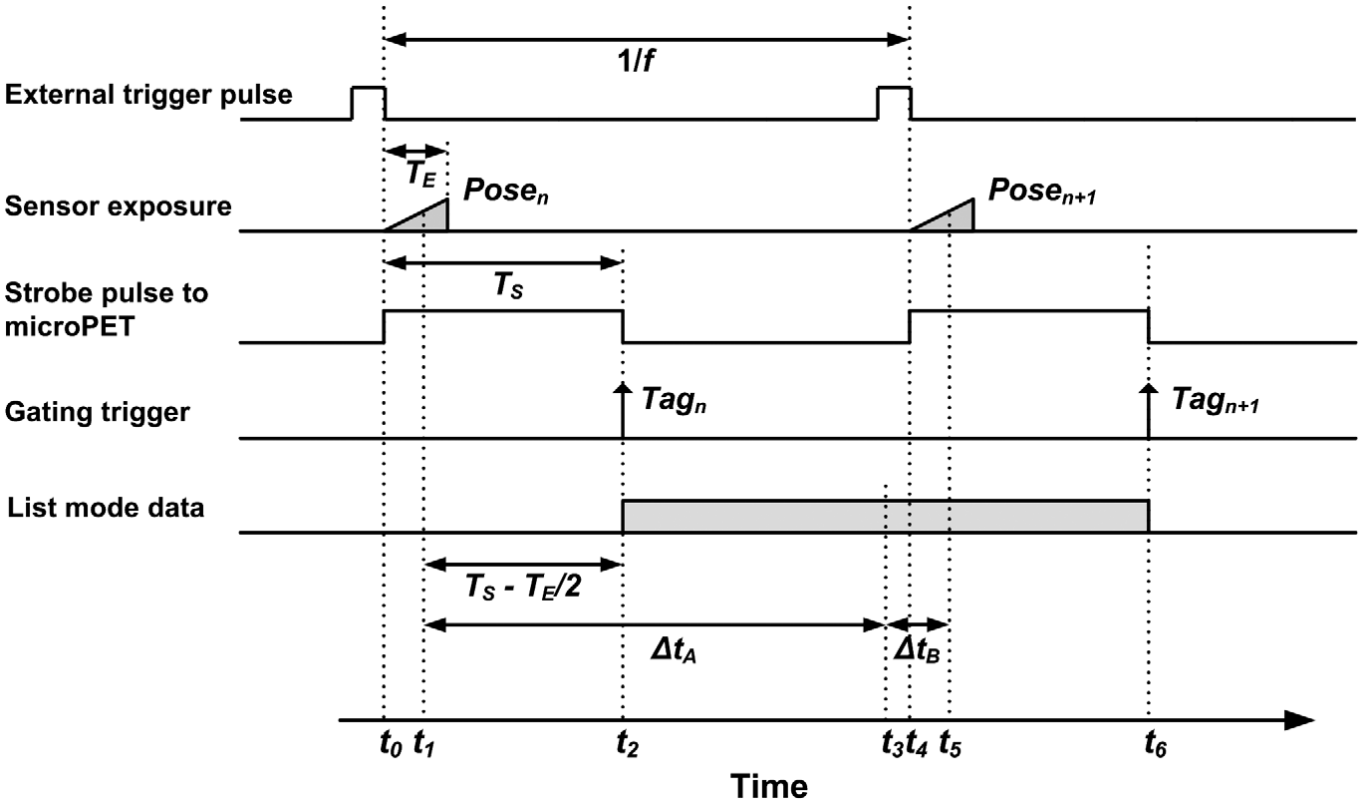
Timing for the synchronisation procedure. The diagram shows the relative timing for key events in the data acquisition and synchronisation processes. Here *f* is the frequency at which the tracker is triggered, *T_E_* is the exposure time for images collected by the cameras, and *T_S_* is the duration of pulses strobed by the tracker when each frame commences. Synchronisation is achieved by matching list mode data segments between consecutive synchronisation tags (eg. shaded rectangle shown between *Tag_n_* and *Tag_n+1_*) with a pose. For the list mode segment between *Tag_n_* and *Tag_n+1_, Pose_n+1_* was the best temporal match.

For motion correction we require each pose measurement used to transform events to be temporally aligned with the list mode data segment to which it is applied. In order to simplify the implementation we chose these segments to be between consecutive synchronisation tags (shaded rectangle in figure 5), taking the time associated with a segment to coincide with the middle of the segment (*t_3_* in figure 5) and the time associated with a pose to coincide with the middle of sensor exposure (*t_1_* and *t_5_* in figure 5). In our experiments, parameter settings were: *T_E_* = 4 ms (sensor exposure), *f* = 30 or 48 Hz (pose triggering frequency) and *T_S_* = 15 ms (strobe pulse width). Three 100 watt incandescent light globes were used as additional lighting to the fluorescent laboratory lights to enable the fast shutter time. The strobe pulse width was >10 ms based on gate input specifications.

In figure 5, *Pose_n+1_*, measured at time *t_5_*, is a better temporal match with the list mode data segment between *Tag_n_* and *Tag_n+1_* than *Pose_n_*, measured at time *t_1_*. Therefore, for the segment of list mode data defined by the tags *Tag_n_* and *Tag_n+1_*, *Pose_n+1_* was used as the best transformation to apply.

### 5. Motion Correction

The list mode data were corrected for motion using the LOR rebinning technique to obtain a normalised motion-corrected sinogram [17], [23], [24]. In this approach, each measured LOR is transformed in space according to the motion measured since the start of the scan (figure 1). Normalisation takes into account the LOR sensitivity for both the measured and transformed LORs so as not to introduce bias [24]. Dead time correction was based on a global scaling factor estimated from statistics extracted from the list mode data as described in [20]. Sinograms were reduced to a series of 2D parallel-plane projections using Fourier rebinning (FORE) [25] and reconstructed using ordered-subsets expectation-maximisation (OSEM) [26] with 16 subsets and 4 iterations. The exception was the reconstruction of the 2-compartment phantom (see below) where the much slower 3D reprojection method (3DRP) [27] was used instead of FORE/OSEM to achieve maximum quantitative accuracy. No apodization filter was used in conjunction with 3DRP. All reconstructed images were corrected for attenuation and scatter and comprised 128×128×95 voxels of size 0.633×0.633×0.796 mm^3^.

### 6. Phantom Studies

Two types of phantom experiment were performed to investigate the ability of this motion tracking and correction methodology to correct for arbitrary continuous motion during PET data acquisition. In all phantom experiments both the 3-point and 8-point markers were fixed firmly to the end of the respective phantom (facing the tracker) for pose tracking.

#### 6.1. Manually applied motion

To investigate the impact of the rate of motion and marker size on tracking accuracy, arbitrary movements of differing rates were manually applied to a Micro Deluxe hot rod phantom (Data Spectrum Corporation, NC, USA). The phantom had an internal diameter of 40 mm and an insert comprising rods with internal diameters 4.2, 4.0, 3.2, 2.4, 1.6 and 1.2 mm.

The hot rod phantom was filled with 17 MBq ^18^F in solution and two separate emission scans were performed on the PET scanner: A 20-min scan during which the phantom was moved steadily and continuously by hand (with the exception of six evenly spaced 1 min intervals when it was kept stationary to simulate periods of relative inactivity of a subject), and a 5-min scan during which the phantom was moved rapidly and continuously by hand. In both cases the movement involved six degrees-of-freedom (DoF) and was done in a roughly oscillatory manner. We attempted to keep the range of motion in each experiment similar. Motion data were collected at 30 Hz. Finally, a 20 min photon transmission scan and a 20 min emission scan were collected, both on the stationary phantom. These provided the necessary data for photon attenuation correction and a motion-free reference, respectively. Transmission data were collected in singles mode using a rotating ^57^Co point source and 110–135 keV energy window.

The list mode data for the motion scans were corrected for motion using the LOR rebinning software and sorted into normalised 3D sinograms before reconstructing them using OSEM as described above. Both motion scans were corrected to a common reference position which was in alignment with the transmission scan (to be used for attenuation correction) and the reference (motion-free) scan.

#### 6.2. Rat head motion

Head movements recorded in an awake rat over a period of 11 min were applied to the hot rod phantom by a six DoF robot manipulator (Epson C3-A601S 6-axis, SEIKO Corp., Japan). Repeatability of the robot was ±20 µm. The study was repeated using a compartment phantom suited to quantitative analyses. It consisted of a main cylindrical compartment with internal diameter 40 mm, and two cylindrical compartments of internal diameter 12 mm within the main compartment. The aims of the robot experiments were to test the feasibility of obtaining accurate motion-corrected images in the presence of realistic rat head motion, and to determine the impact of varying the pose sampling rate and synchronisation accuracy.

The rat head motion data were obtained in accordance with a protocol approved by the Animal Ethics Committee of the University of Sydney (Protocol Number: K00/12-2008/2/4891). A single male Sprague Dawley rat, 14 wks old, was group housed in a Plexiglas cage, two animals per cage. It was maintained in ambient temperature (22–24°C) on a 12∶12 light:dark cycle. Food and water were provided ad libitum.

To obtain head motion data the animal was positioned in a PVC tube (60 mm diameter) inside the microPET with its head protruding from the end. The tube was supported by the scanner bed which was rigidly mounted at the rear of the scanner so that the tracker could occupy the usual bed support (see Materials & Methods section 3). Tracking followed 10 days, 20 min/day, of acclimatisation to the scanner environment and tube. A 3-point marker was glued to the rat's forehead and motion data were collected at 30 Hz for a period of 40 min using the MicronTracker.

**Figure 6:**
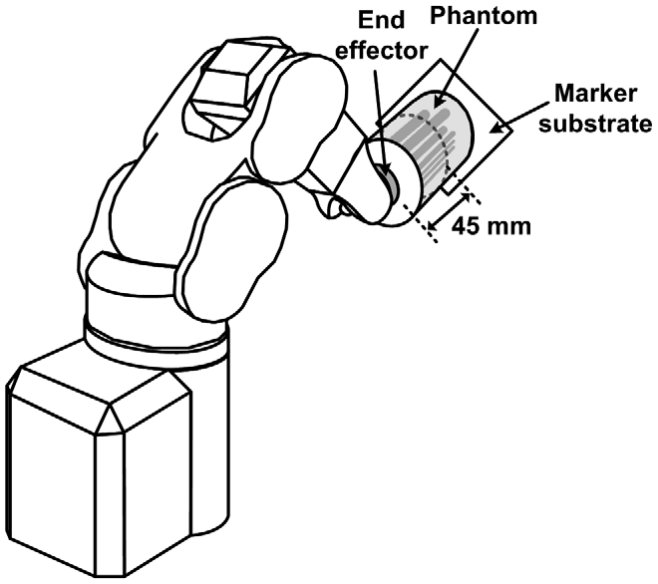
Setup for the robot-controlled motion. Diagram showing the six degree-of-freedom robot with the hot rod phantom attached to the end-effector. The apparent location of the end-effector was shifted along the axis of the phantom by 45 mm, corresponding to the middle third of the cylinder. This more closely resembled the proximity of the marker to the brain in the original motion data. The 8-point and 3-point markers used for tracking were stuck to the marker substrate (on the opposite side to that visible here).

A calibration, similar to the one performed for the tracker and scanner, was performed to convert between the tracker and robot coordinate systems. Marker poses in robot coordinates were converted to movements relative to the initial pose and the trace smoothed using a moving average implementation of the pose averaging method in [28]. The smoothed movements, **T**_M*i*_, were then converted to the commanded robot poses according to:
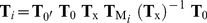
(1)


where **T** *_i_* represents the *i*-th commanded pose streamed to the robot, **T**_x_ is a transformation matrix to adjust the location of the end-effector, and **T**_0_ and **T**_0'_ represent the pose of the robot at calibration and at the start of the experiment, respectively, both of which enabled the required movements to be performed from the arbitrary robot starting pose **T**_0'_. The 3-point marker used to measure the original head motion was a few millimetres from the brain; to apply similar motion to the middle third of the phantom it was necessary to shift the apparent end-effector location by 45 mm towards the centre of the phantom using **T**_x_ (see figure 6).

The first 20,000 poses (representing 11 min of rat motion) were streamed to the robot motion controller in remote control mode using a TCP/IP connection established by third-party software (Visual Basic, Microsoft Corp., USA). The maximum angular speed and angular acceleration parameters for the robot end-effector were 750 deg.s^−1^ and 4450 deg.s^−2^, respectively, and were chosen so that all the movements were completed in 660 s. This gave an average time per pose of 33 ms, identical to the measured rat data.

One internal cylinder and the outer compartment of the 2-compartment phantom were filled with a 3∶1 ratio of known concentrations (given below) of ^18^F in water, respectively, and the remaining internal cylinder was filled with water. The hot rod phantom was filled with 20 MBq of ^18^F. Each phantom was attached to the robot end-effector in turn using a custom-made Perspex™ adaptor and scanned for 12 min, during 11 min of which it underwent the pre-programmed rat movements. The phantom was stationary for the initial and final 30 s of the scan. Each phantom was scanned twice, once using 30 Hz pose sampling and once using 48 Hz. A 20 min photon transmission scan for attenuation correction and a 30 min reference (motion-free) emission scan were also collected for each phantom. The concentrations of ^18^F in the hot cylinder of the compartment phantom during the 30 Hz, 48 Hz and reference scans were 1.3 MBq.mL^−1^, 1.1 MBq.mL^−1^ and 0.95 MBq.mL^−1^, respectively. These values reflect typical striatal concentrations of [^18^F]FDG in rat brain studies for an injected dose of 50 MBq (e.g. [29]). Motion correction and reconstruction were performed similarly to the manual motion experiments described above except that the 3DRP algorithm was used to reconstruct the compartment phantom data for the bias analysis, described below.

In order to explore the impact of pose sampling rate and system synchronisation error on the motion-corrected images, the 48 Hz motion data were manipulated in two ways: (i) down-sampled (by neglecting intermediate pose measurements) to simulate sampling rates of 48, 24, 16, 12, 9.6, 4, 2, 1, 0.5 and 0.25 Hz and (ii) time-shifted by varying amounts to simulate constant synchronisation errors of ±0.1, ±0.2, ±0.3, ±0.4, ±0.5, ±0.6, ±0.7, ±0.85, ±1 and ±2 s. In each case the motion data were used for motion correction, including attenuation and scatter correction, and the bias for the hot cylinder in the compartment phantom was computed as
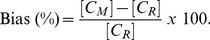
(2)


Here, the subscripts *M* and *R* refer to the motion-corrected and reference concentrations, respectively. Concentrations were computed from regions drawn in the middle third of the phantom compartments and the reference concentration was obtained from the motion-free reconstruction.

### 7. Motion Analysis

Measured pose data acquired in the phantom experiments were analysed to determine the range and rate of motion with respect to the scanner axes. For position given by **P** = [*P_x_*, *P_y_*, *P_z_*] and rotation given by the 3x3 orthonormal rotation matrix **R** (where **R** can equally be expressed as three ordered Euler rotation angles *α_x_*, *α_y_* and *α_z_* about the *x*, *y* and *z* axes, respectively), the sample-wise rates of change were calculated according to:
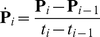
(3a)

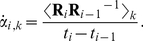
(3b)


Here *i* is the sample number, *t_i_* refers to the time of the *i*-th sample, and the bracket notation in the numerator of (3b) indicates the component of rotation about either the *x*, *y* or *z* axis with *k* identifying the particular axis. The rates were calculated after applying a median filter (width 5 samples) to the data to remove occasional outliers. Equation (3a) represents the linear velocity of the marker origin during the experiment, dependent on both the rotations and the physical location of the marker. This parameter is important from a motion tracking perspective because it relates to the motion blur expected for a given shutter speed. In all of our phantom experiments the marker was positioned identically on the phantom and therefore the ranges and rates reported were directly comparable.

### 8. Awake Animal Study

Feasibility of our motion tracking and correction approach was tested in a live animal study. The animal described in Materials & Methods section 6.2 was injected with approximately 80 MBq of ^18^F-FDG via the tail vein while under isoflurane/O_2_ gas anaesthesia (1.5%). Following a 20-min uptake period the head was scanned in the microPET for 20 min, during which the head pose was sampled at 30 Hz by the MicronTracker. Motion correction was applied as described in Materials & Methods section 5. The corrected data were then reconstructed using OSEM. Attenuation and scatter corrections, based on a calculated attenuation map derived from this reconstruction, were included.

## Results

### 1. Manually Applied Motion

**Figure 7:**
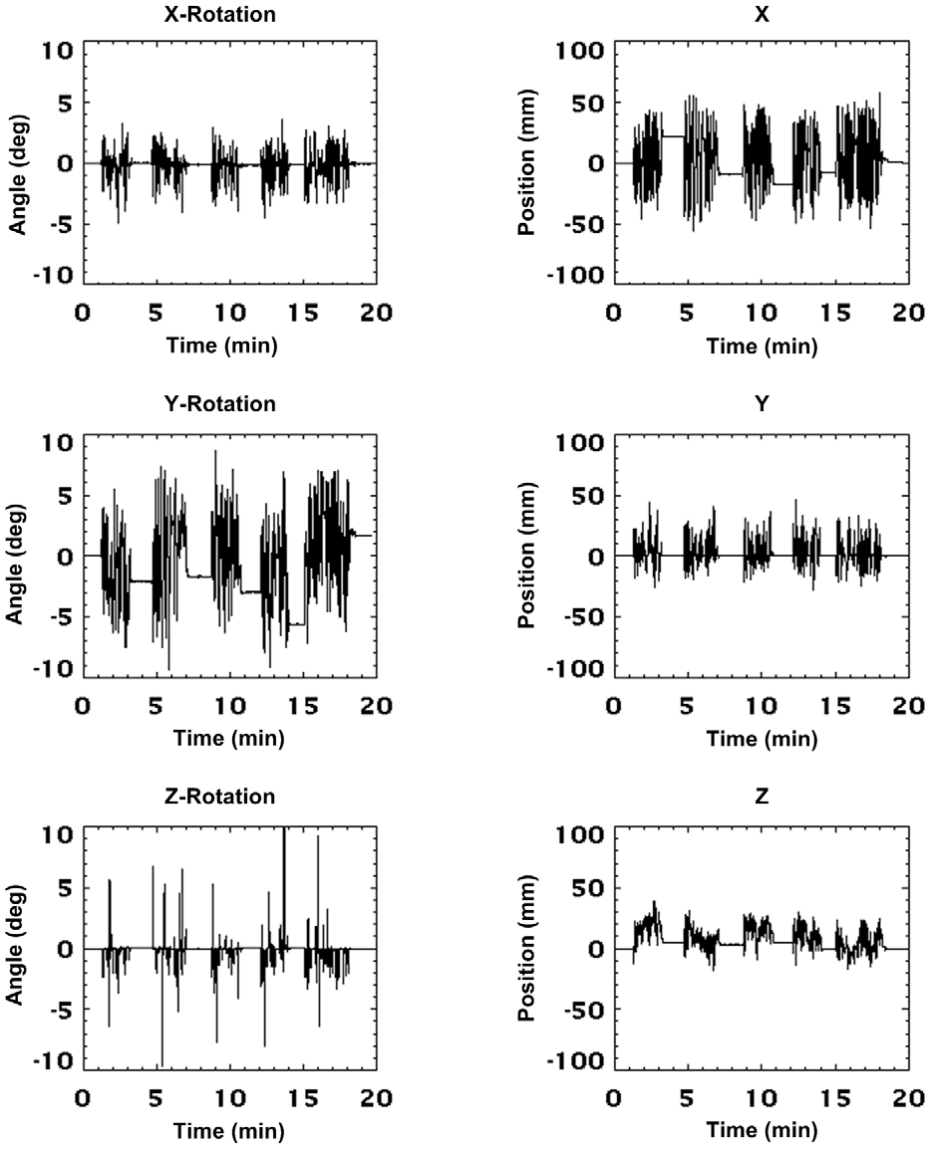
Manually applied motion (‘slow-moderate’). Motion data recorded for each degree-of-freedom during the 20-min phantom study involving slow-moderate manually applied motion. Continuous, arbitrary motion was applied for 2–3 min intervals, interspersed by approximately 1 min intervals when the phantom was kept stationary. Data are shown in the scanner coordinate system and represent the cumulative motion since the start of the scan. The pose sampling rate during tracking was 30 Hz.

**Figure 8:**
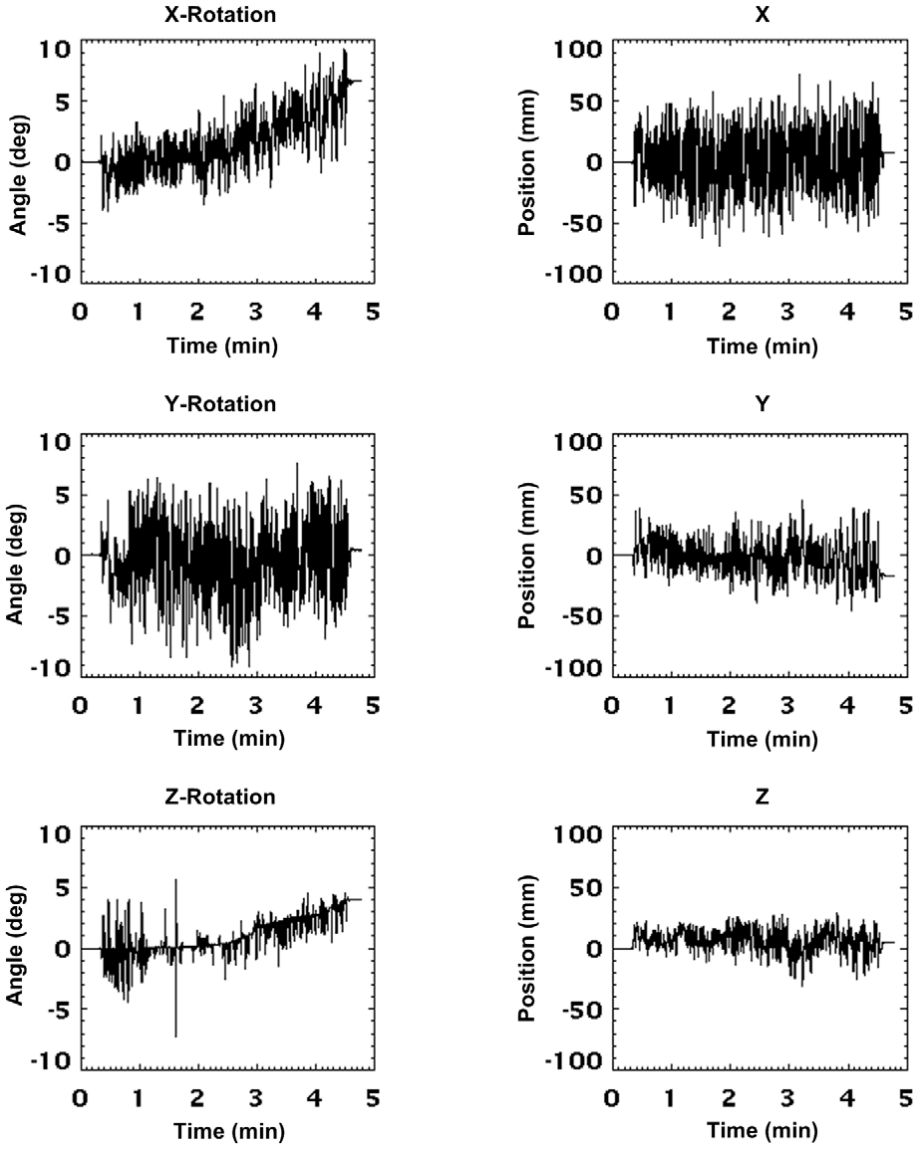
Manually applied motion (‘fast’). Motion data recorded in each degree-of-freedom during the 5-min phantom study involving fast manually applied motion. Continuous, arbitrary motion was applied for approximately 4.5 minutes out of the total scan time. Data are shown in the scanner coordinate system and represent the cumulative motion since the start of the scan. The pose sampling rate during tracking was 30 Hz.

**Figure 9:**
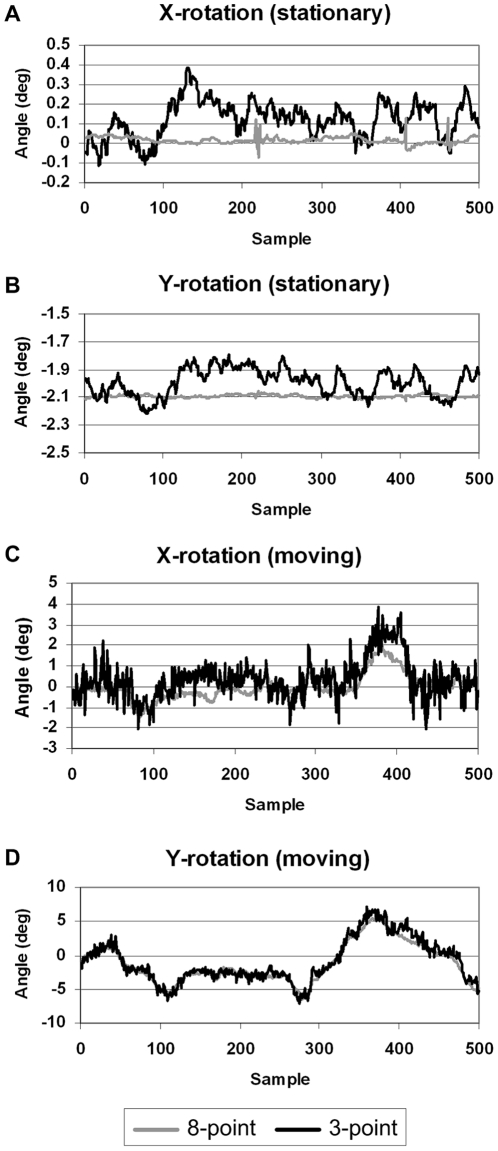
Measurement jitter for motion tracking markers. Comparison of the measurement jitter in typical segments (15–20 s duration) of motion data obtained from the 3-point marker (black line) and 8-point marker (grey line) during the first 5 minutes of the 20 min manually applied motion study (slow-moderate motion). (a, b) Measured rotation about the *x* and *y* axes, respectively, when the phantom was stationary; (c, d) measured rotation about the *x* and *y* axes, respectively, when the phantom was moving. Rotations about *x* and *y* are shown because they exhibited the greatest measurement jitter. Note that these data are in scanner coordinates and that sample numbers are relative to the start of the selected segments. The pose sampling rate during tracking was 30 Hz.

The motion of the phantom during the 20 min and 5 min scans is shown in figures 7 and 8, respectively. Data are in microPET scanner coordinates and represent the cumulative motion relative to the start of the scan. The figures show the continuous nature of the applied motion and the higher frequency of motion during the 5 min scan. The flat sections in figure 7 correspond to the 1-min rest intervals in which the phantom was stationary. Figure 9 shows typical measurement jitter for the two different marker sizes when stationary and moving. Data are shown for rotation about the *x* and *y* axes since these DoFs exhibited the greatest amount of jitter. The 3-point marker data deviated from the 8-point marker data by up to 0.4° in the stationary case and 3° in the moving case. Note that the reduced jitter for stationary markers results from a moving average filter that is automatically applied in software when a marker is detected to be stationary. This filter is off when a marker is moving.

Tables 1 and 2 summarise the absolute range and rate of motion for each DoF for the 20 min and 5 min scans, respectively. The maximum range of motion was comparable for both studies: *x*, *y* and *z* rotations varied from 10–30°, and translations varied from 50–150 mm. About 75% of samples were within +/−10° and +/−60 mm. In contrast, the maximum absolute rates of rotational and translational motion were 2–3 times greater for the fast motion compared to the slow/moderate motion. In terms of the 75th percentile metric (see tables 1 and 2) the fast motion was about 6–8 times faster than the slow/moderate motion.

**Table 1:** Range and rate of motion for the slow-moderate manually applied motion.

	Range of Motion (abs.)^1,2^ (mm, deg)		Rate of Motion (abs.)^1,2^ (mm, deg)	
DoF	Max	75% range	Max	75% level
***x*** **-rot**	10	3	11	1
***y*** **-rot**	19	9	22	2
***z*** **-rot**	28	1	17	0
***x***	114	43	249	15
***y***	74	13	90	9
***z***	57	20	64	6

1 Ranges and rates were computed from the 8-point marker data.

2 All values rounded to the nearest integer.

**Table 2:** Range and rate of motion for the fast manually applied motion.

	Range of Motion (abs.)^1,2^ (mm, deg)		Rate of Motion (abs.)^1,2^ (mm, deg)	
DoF	Max	75% range	Max	75% level
***x*** **-rot**	15	7	35	7
***y*** **-rot**	18	8	52	13
***z*** **-rot**	13	4	41	3
***x***	140	56	496	120
***y***	90	30	264	64
***z***	60	18	194	39

1 Ranges and rates were computed from the 8-point marker data.

2 All values rounded to the nearest integer.

**Figure 10:**
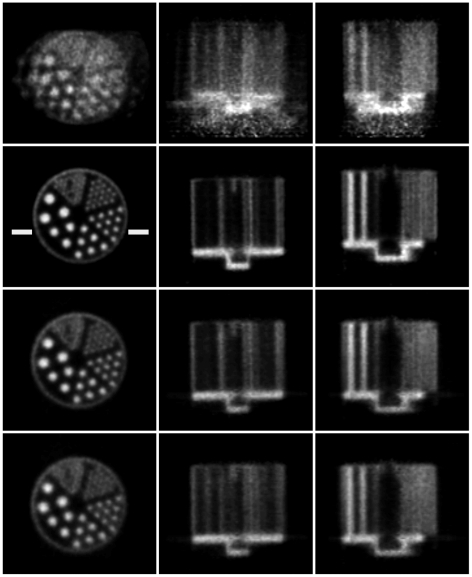
Motion correction of slow-moderate manual motion. Motion correction of the 20-min hot rod study with slow-moderate manually applied motion (see figure 7). Left to right are orthogonal views of the centre of the phantom. Row 1: no motion correction; row 2: motion free; row 3: correction based on the 8-point marker; row 4: correction based on the 3-point marker. The pose sampling rate during tracking was 30 Hz. Note that the white bars marked on the motion-free image represent the location of profiles shown in figure 12.

**Figure 11:**
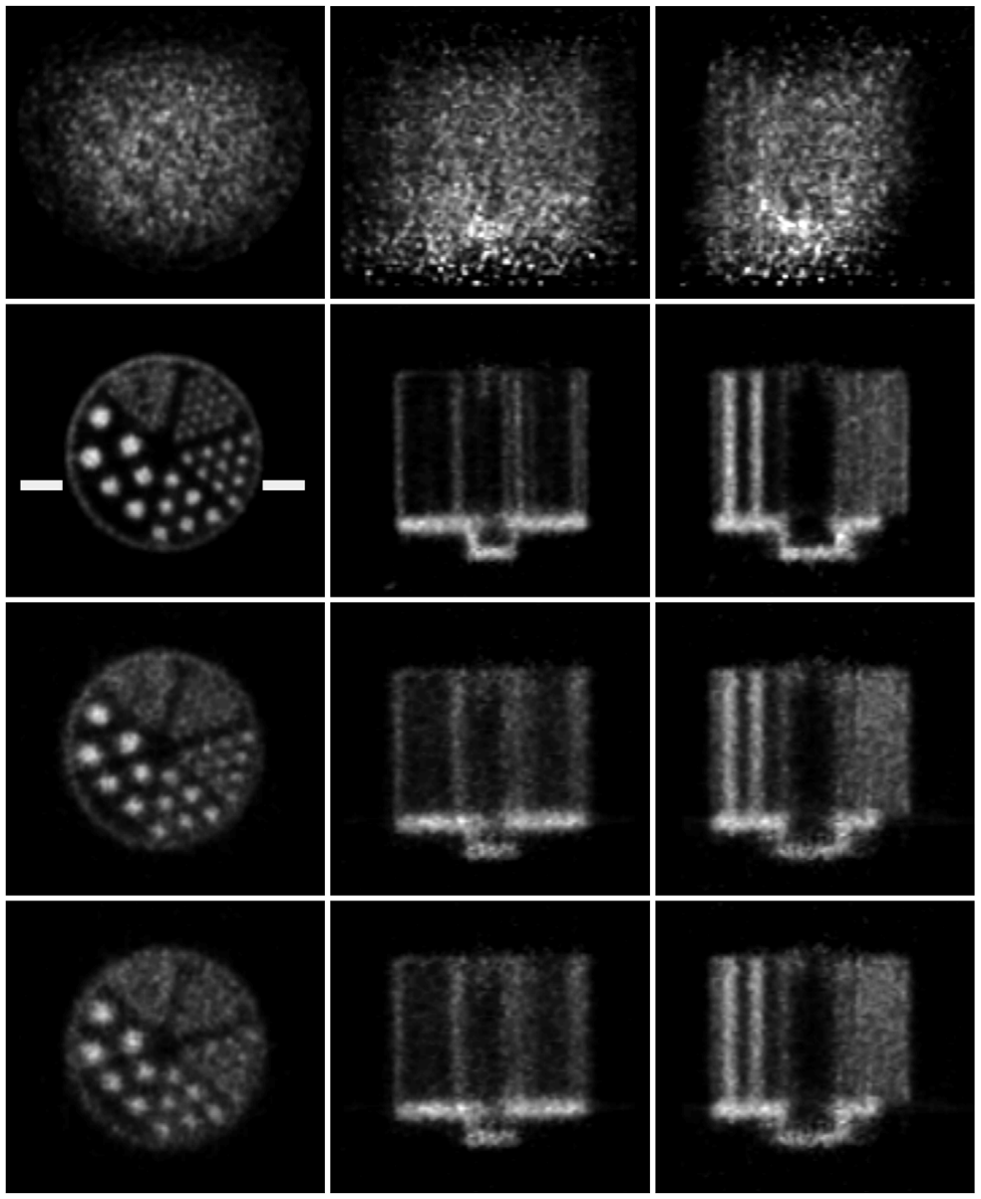
Motion correction of fast manual motion. Motion correction of the 5-min hot rod study with fast manually applied motion (see figure 8). Left to right are orthogonal views through the centre of the phantom. Row 1: no motion correction; row 2: motion free; row 3: correction based on the 8-point marker; row 4: correction based on the 3-point marker. The pose sampling rate during tracking was 30 Hz. Note that the white bars marked on the motion-free image represent the location of profiles shown in figure 12.

Reconstructed slices of the phantom in three orthogonal planes, before and after motion correction, are shown in figures 10 and 11 for the 20 min and 5 min studies, respectively. Qualitatively, in both cases the uncorrected slices showed obvious degradation (for the fast movement (figure 11) no detail of the hot rods was discernable) and the corrected slices showed marked improvement with respect to the motion-free. For the slow and fast motion, respectively, rod diameters of 1.6 mm and 2.4 mm were resolved after correction using both markers, though correction was better in each case using the 8-point marker.

**Figure 12:**
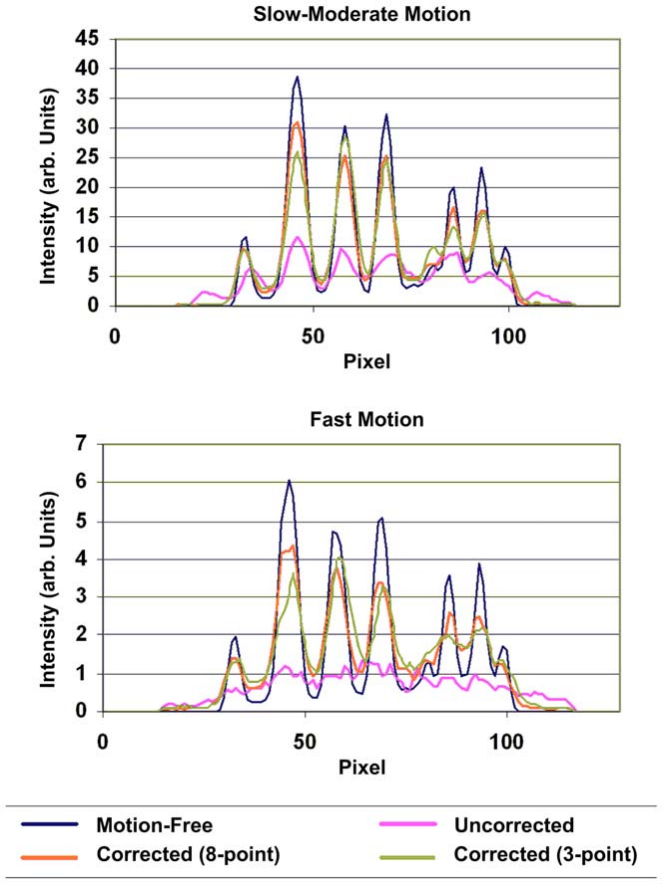
Quantitative assessment of motion correction. Comparison of the reconstructed hot rod phantom images with and without motion correction. The top panel shows profiles for the 20-min study with slow-moderate manually applied motion, and the bottom panel shows profiles for the 5 min study with fast manually applied motion. Profiles were drawn through the transverse reconstructed images at the level indicated in figures 10 and 11. The pose sampling rate during tracking was 30 Hz.

Profiles are shown in figure 12 for the 20 min and 5 min studies. These represent the summed activity of 5 rows for 5 central transverse slices. The level at which profiles were chosen is indicated in figures 10 and 11. Good agreement between the motion-corrected and reference profile was obtained irrespective of which marker was used for tracking but the 8-point marker gave better contrast (larger peak-trough distances) in general. In all cases the uncorrected profile was severely blurred.

**Figure 13:**
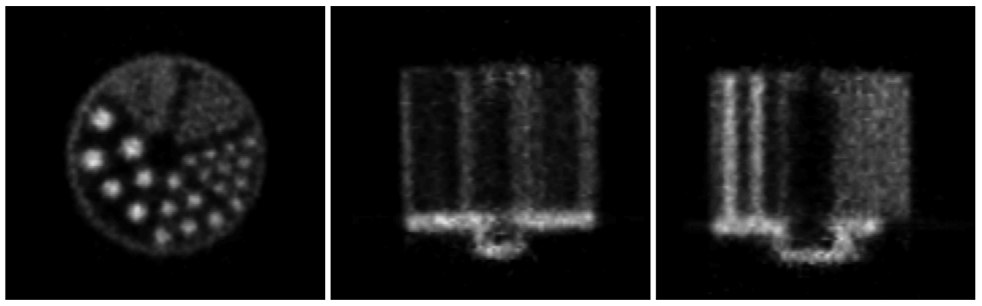
Effect of motion rate on motion correction. Motion correction of the hot rod study with slow-moderate manually applied motion. A 72 s segment of the data has been corrected so that it is comparable (in terms of counting statistics) with the fast motion study. Left to right shows orthogonal views of the centre of the phantom. Correction was based on the 8-point marker measurements. These images can be compared with those in figure 11 (row 3) in order to see the effect that the rate of motion had on motion correction accuracy.

The 20 min and 5 min studies differed primarily in the rate of motion. To better compare the impact of motion rate on motion correction, a 72 s segment of list mode data was extracted from the 20 min study, motion-corrected and reconstructed. This duration was chosen so that the two studies had comparable counting statistics. Motion correction was based on the motion data from the 8-point marker in order to minimise the effect of marker size. The result is shown in figure 13. Comparison of figure 13 with figure 11 (row 3) indicates that increasing the rate of motion resulted in poorer correction, e.g. the 1.6 mm diameter rods were more clearly resolved for the slower motion.

### 2. Robot-Controlled Motion

**Figure 14:**
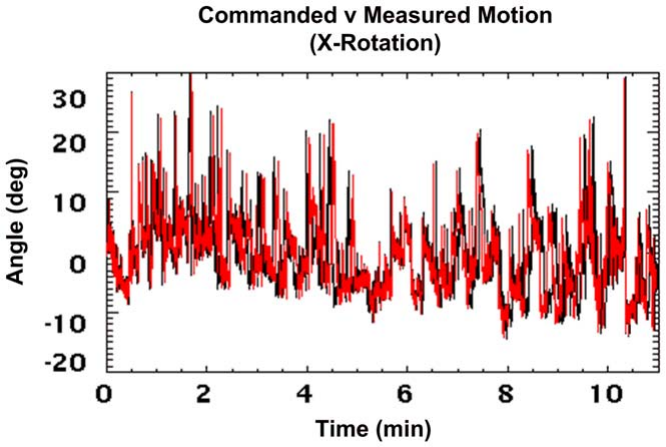
Comparison of commanded and measured robot motion. Commanded robot motion (black) overlaid with measured motion (red). Data are shown for the *x*-axis rotation. Note that there were 20,000 poses spanning 11 min. All measurements are in robot coordinates and represent the cumulative motion since the start of the scan.

Figure 14 shows commanded robot motion, simulating that of an awake rat, overlaid with measured motion collected at 30 Hz for the *x*-axis rotation. Data have been converted to robot coordinates and represent the cumulative motion relative to the start of the scan. The close agreement of the curves indicates that the tracker faithfully executed the commanded motion. Occasional temporal misalignment of the curves was due to the variable time taken by the robot to assume each new pose - dependent on the change in pose, the path calculated by the controller, and the preset speed and acceleration limits. However, the average time per executed pose was 33 ms, the same as for the commanded motion.

Table 3 summarises the absolute range and rate of motion of the hot rod phantom for each DoF. Compared to the fast manually applied motion (table 2), the angular ranges were consistently higher and the translational ranges were comparable. Angular rates were also comparable whereas the translational rates were comparable to those for the slower manually applied motion.

**Table 3:** Range and rate of motion for the robot-generated motion.

	Range of Motion (abs.)^1,2^ (mm, deg)		Rate of Motion (abs.)^1,2^ (mm, deg)	
DoF	Max	75% range	Max	75% level
***x*** **-rot**	40	15	63	11
***y*** **-rot**	29	17	57	8
***z*** **-rot**	91	38	88	3
***x***	51	30	86	6
***y***	47	16	72	9
***z***	11	4	26	3

1 Ranges and rates were computed from the 8-point marker data.

2 All values rounded to the nearest integer.

**Figure 15:**
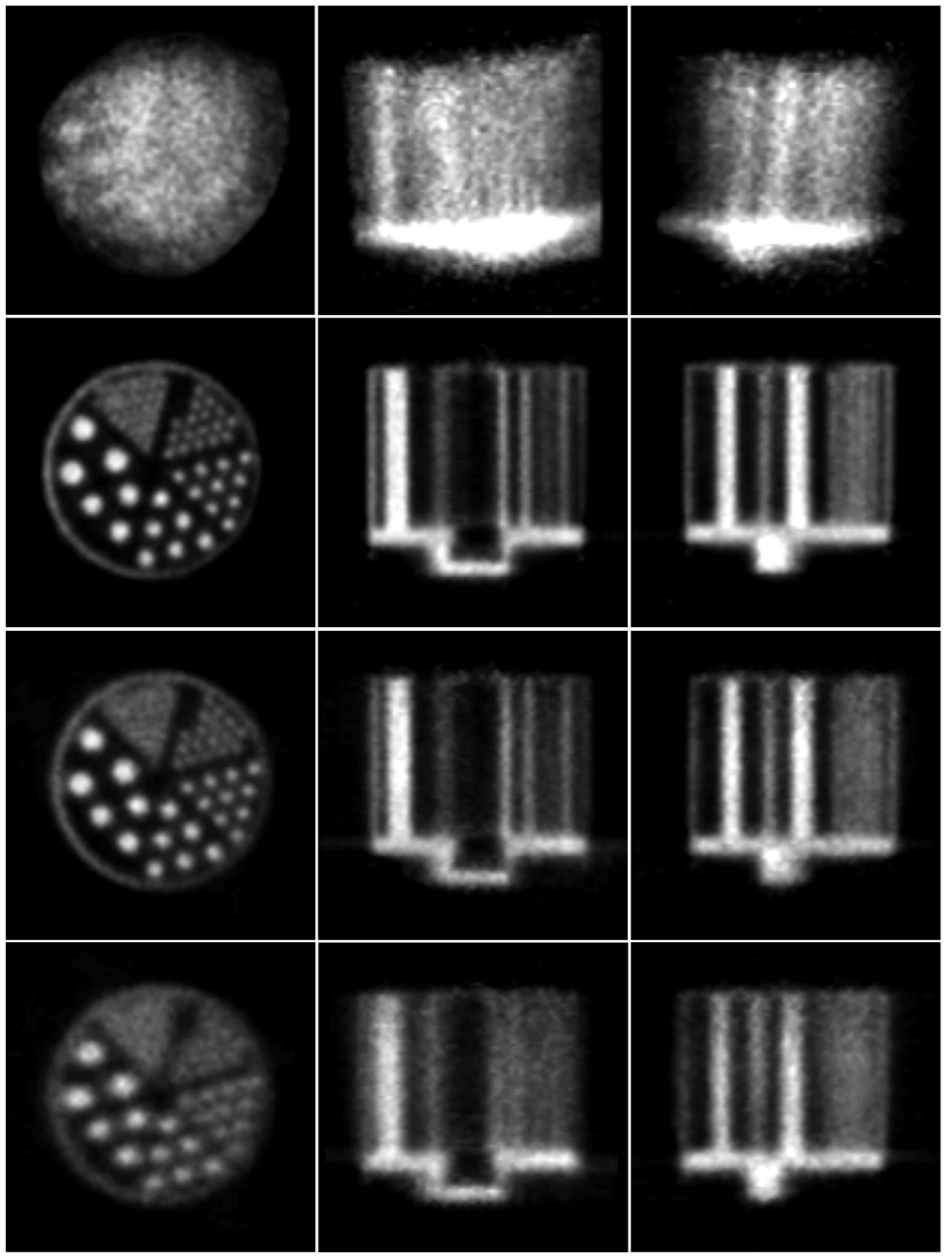
Motion correction of simulated rat motion (hot rod phantom). Motion correction of the hot rod study corrupted by the robot-generated rat motion. Left to right are orthogonal views of the centre of the phantom. Row 1: no motion correction; row 2: motion free; row 3: correction based on the 8-point marker; row 4: correction based on the 3-point marker. The pose sampling rate during tracking was 30 Hz.

Reconstructed slices of the hot rod phantom for the rat motion are shown in figure 15. Correction based on 8-point marker tracking resulted in excellent agreement with the motion-free reconstruction, 1.6 mm diameter rods being clearly resolved. Correction based on 3-point marker tracking was noticeably inferior as only the 2.4 mm diameter rods were resolved; this gave results similar to those obtained for the fast manually applied motion (figure 11).

**Figure 16:**
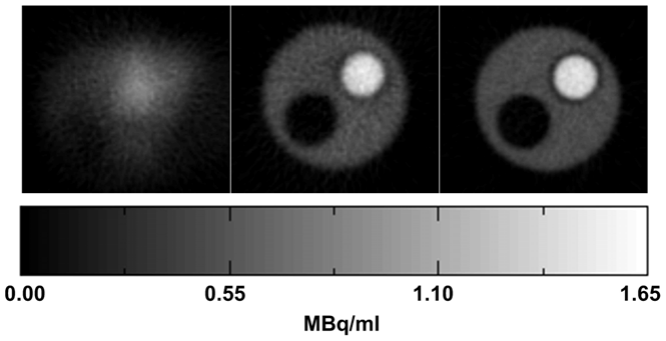
Motion correction of simulated rat motion (2-compartment phantom). Central transverse slice of the compartment phantom shown for the uncorrected (left), motion-corrected (middle) and motion-free (right) reconstructions. Correction was based on the 8-point marker data. The hot (1.3 MBq.ml^−1^), cold (0 MBq.ml^−1^) and background (0.43 MBq.ml^−1^) compartments of the phantom were clearly visible in the corrected and motion-free reconstructions. The pose sampling rate during tracking was 30 Hz.

Figure 16 shows transverse reconstructed slices of the compartment phantom, before and after motion correction based on the 8-point marker, together with the motion-free reconstruction. As in the earlier experiments, the degrading effects of the rat motion and the improvement after motion correction were evident. The less noisy appearance of the reference images is due mainly to the increased counting statistics in this study.

**Figure 17:**
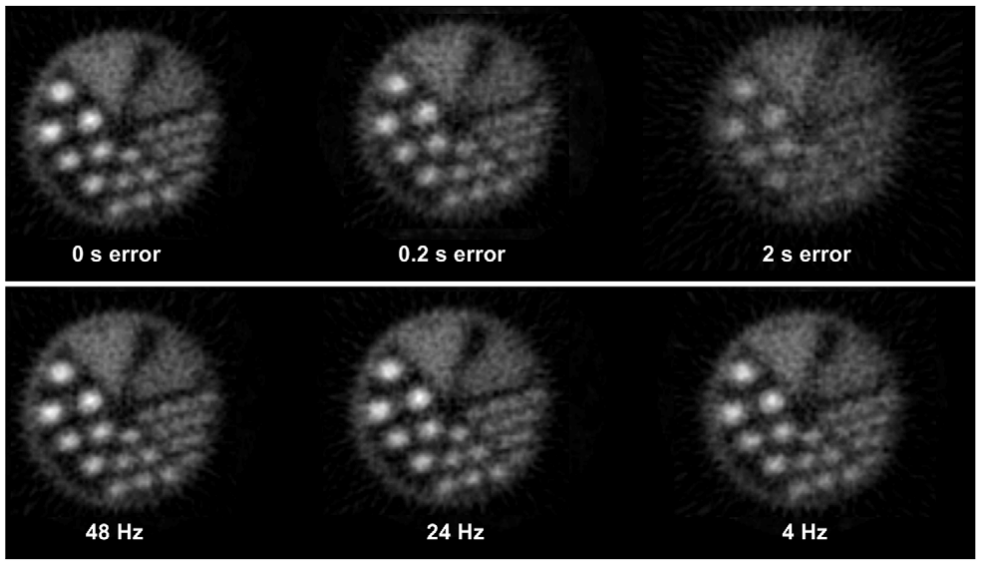
Effect of synchronisation error and pose sampling rate on motion correction. Examples of motion-corrected hot rod phantom reconstructions obtained with varying degrees of tracker-scanner synchronisation error (top row) and various pose sampling rates (bottom row). Results are shown for a central transverse slice.

**Figure 18:**
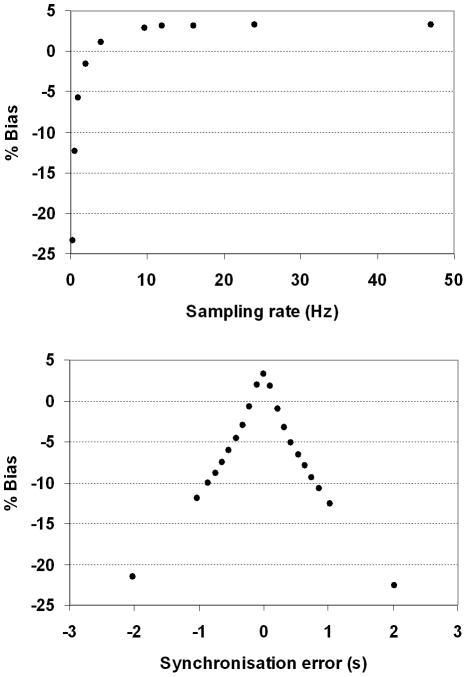
Quantitative performance of PET as a function of synchronisation error and pose sampling rate. Bias (%) in concentration for the hot cylinder of the 2-compartment phantom as a function of pose sampling rate (top) and synchronisation error (bottom). The simulated pose sampling rates ranged from 0.25 to 48 Hz and the simulated synchronisation error ranged from −2 to 2 s.

The effect of changing the pose sampling rate and synchronisation error are shown in figures 17 and 18. Both sets of results are based on the 3-point marker, suitable for an animal. Figure 17 shows how these two variables affected visual image quality for the hot rod phantom. In each case the same central transverse slice is shown. Synchronisation error of 0.2 s produced noticeable degradation of the image whereas only minor degradation was apparent for sampling rates above 20 Hz. Figure 18 shows the effect on bias for the hot cylinder of the compartment phantom. Bias at the maximum sampling rate (48 Hz) with no added synchronisation error was approximately +3 %. This residual error is likely to be due, in part, to the global correction factor used to compensate for lost events during LOR rebinning. LORs that are spatially transformed in the rebinning process such that they no longer intersect with the detector rings are referred to as ‘lost’ events. The effect can be approximately compensated for by applying a global scaling factor to the reconstruction [30]. The upper plot in figure 18 shows that bias worsened most rapidly at pose sampling frequencies below approximately 10 Hz but was relatively stable above approximately 20 Hz. Moreover, the lower plot in figure 18 shows that bias worsened rapidly with increasing synchronisation error in either direction. A greater than 10% change in the bias resulted from synchronisation errors in excess of 0.6 s.

**Figure 19:**
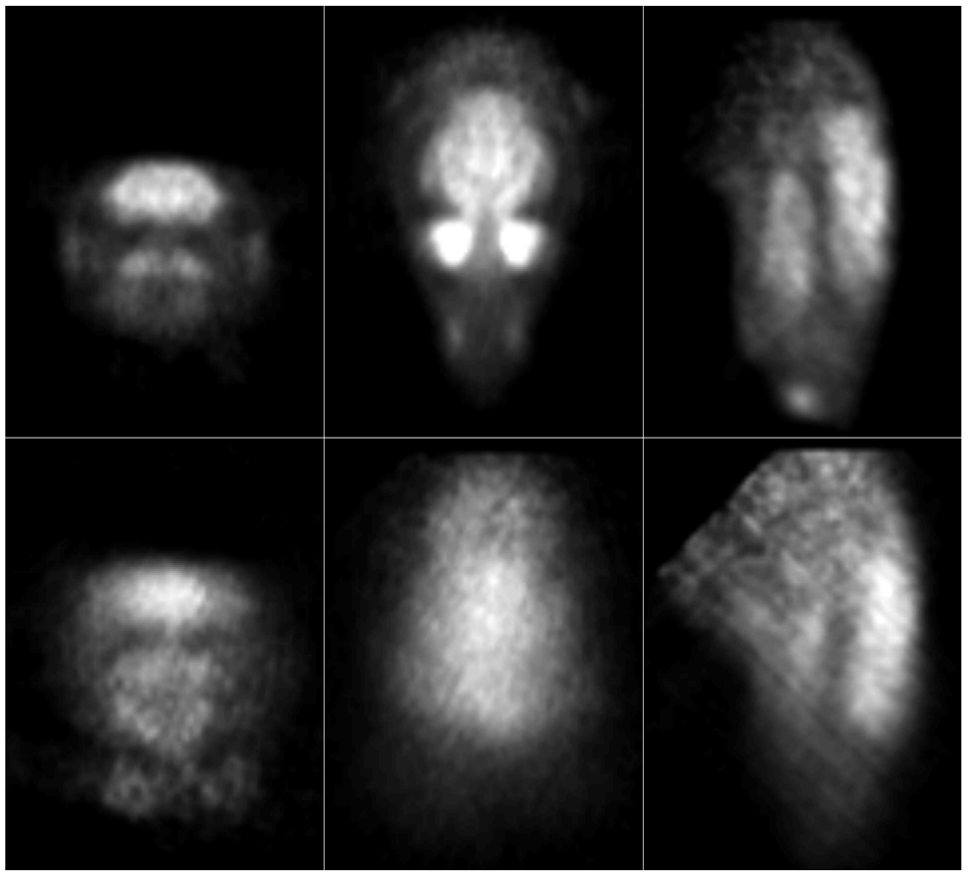
Motion correction applied to an awake rat study. Orthogonal reconstructed slices for the corrected (top row) and uncorrected (bottom row) study.

Figure 19 shows orthogonal slices obtained from the uncorrected and motion-corrected animal data. We stress that in this case no reference measurement was available for comparison. However, as in the phantom studies, it was clear that, qualitatively, there was a marked improvement after motion correction. This indicates the feasibility of using our methods in conjunction with a small marker for motion correction in the intended application.

## Discussion

We have explored factors affecting motion tracking accuracy, which in turn could affect downstream motion compensation and reconstruction accuracy in PET studies of awake rats. Results from phantom experiments demonstrate that the hardware-based synchronisation scheme enables robust correction of rapid and continuous arbitrary rigid-body motion, including realistic rat head motion. Our data support the conclusion that synchronisation error and sampling rate are critical parameters to optimise for a motion tracking system aimed at compensating for a typical rat head motion pattern. A pose sampling rate in excess of 20 Hz and synchronisation accuracy within 100 ms appear necessary to achieve quantitatively acceptable results (<5 % error) in awake rat studies. We have also demonstrated dependencies of motion tracking accuracy on the marker size and object speed. Although we show that a large marker about 60 mm across provides greater tracking accuracy than a smaller one, effective motion compensation can still be achieved using a miniature marker suitable for attachment to a rat's head. In practice, the accuracy of tracking will also depend to some extent on the velocity of motion, as demonstrated by our noise and motion analysis results.

Results from the rat study demonstrate the feasibility of using this motion tracking and correction approach in the intended application. However in the present work we have based conclusions regarding quantitative accuracy on phantom studies rather than live subjects in order to have a directly comparable motion-free reference scan. Anaesthetic and tracer washout effects, which alter the tracer distribution, prevented use of the same animal as a gold standard.

In the awake animal, it is also recognised that, although the head is treated as a rigid body, not all parts of the head move in the same way – for example, the lower jaw and neck. For events originating from these regions, only an approximate correction can be obtained using the motion data. The resultant mis-positioning of these events does not appear to significantly contaminate the brain signal.

In the ‘back-to-front’ matching (synchronisation) described in the methods, we chose to simplify the implementation by assigning poses to segments of the list mode data that fell between consecutive synchronisation tags. Based on this, the timing diagram in figure 5 indicates that the synchronisation error, Δ*t_2_*, is dependent on the sampling frequency, *f*, and the strobe pulse width, *T_S_*. For our phantom experiments we chose *T_S_* to be constant (15 ms), giving |Δ*t_2_*| = 3.5 ms for the motion experiments ( *f* = 30 Hz) and |Δ*t_2_*| = 8.5 ms for the sampling frequency and synchronisation error simulations ( *f* = 48 Hz). The temporal alignment of data streams we achieved in these experiments was therefore <10 ms. However, it is clear that *T_S_* (and *f* ) could be chosen for any particular experiment to make Δ*t_2_* smaller. In practice, there are certain constraints that may preclude reducing Δ*t_2_* to zero. One example is the requirement that *T_S_* be greater than 10 ms, a value corresponding to half the duty cycle of the 50 Hz maximum trigger rating specified for the scanner gating input. Alternatively it is possible, with a relatively minor modification to the present software, to choose the segments of list mode data to which transformations are applied such that the synchronisation error is always reduced to approximately zero. We intend to investigate whether doing this provides any further improvement in motion correction accuracy.

Marker size clearly plays a major role in motion tracking accuracy and, as expected, the smaller marker gave less accurate results in our experiments [22], [23]. This was particularly noticeable for the simulated rat motion (figure 15). We suspect this is due in part to vibration of the robot end-effector when performing many small movements at high acceleration, and that jitter noise for the smaller marker may have been exacerbated by this vibration. In spite of this, the small marker was a suitable size for rat head tracking and our results demonstrate that effective correction of rapid motion can be achieved when it is used. In future work we will try to optimise tracking accuracy for markers of this size through the use of filtering techniques (eg. [31]).

Object speed, although not controllable, is an important factor dictating motion tracking requirements. In our experiments the maximum linear speed of the marker origin was 0.5 m.s^−1^ (for the fast manually applied motion). Given the small angular motions in this case (<13°), 0.5 m.s^−1^ is a reasonable estimate of how quickly individual voxels could be tracked and corrected using this tracking system with 30 Hz sampling. An analysis of the speed of voxels of interest in live subjects is likely to shed more light on speed-related motion tracking requirements for this application and will be the subject of future work.

Recently, regional neurochemical changes in the brain, temporally correlated with behavioural changes, were demonstrated in awake, unrestrained rats using a miniature, head-mounted PET tomograph (RatCAP) secured to the animal's head and counter-balanced [15]. In spite of the limitations of the counterbalance mechanism, the RatCAP allows the animal to be relatively unrestrained – a situation which is challenging to reproduce in a conventional scanner. However, it requires a surgical procedure and acclimatisation of the animal to the apparatus. Further, due to size and weight restrictions the detection efficiency of the RatCAP is relatively low compared with a conventional animal PET system, resulting in poor signal-to-noise ratio [32].

By comparison, the approach described here is non-invasive, and can utilise conventional microPET scanners with high detection efficiency for improved signal-to-noise. It would also appear to be more readily scalable to mice than the RatCAP [16]. Overall the results presented indicate the importance of optimised motion tracking for quantitatively accurate motion-corrected PET imaging of awake animals, and that motion tracking parameters for effective motion correction in preclinical brain imaging of awake rats are achievable in the laboratory setting. This could broaden the scope of animal experiments currently possible with PET.
